# FMRP, a multifunctional RNA-binding protein in quest of a new identity

**DOI:** 10.3389/fgene.2022.976480

**Published:** 2022-08-10

**Authors:** Edouard W. Khandjian, Claude Robert, Laetitia Davidovic

**Affiliations:** ^1^ Centre de Recherche CERVO, Département de Psychiatrie et de Neurosciences, Faculté de Médecine, Université Laval, Québec City, QC, Canada; ^2^ Département des Sciences Animales, Faculté des Sciences de l’agriculture et de l’alimentation, Université Laval, Québec City, QC, Canada; ^3^ Centre de Recherche en Reproduction, Développement et Santé Intergénérationnelle (CRDSI), Université Laval, Québec City, QC, Canada; ^4^ Université Côte d’Azur, CNRS, Institut de Pharmacologie Moléculaire et Cellulaire, Valbonne, France

**Keywords:** FMR1, FMRP, fragile X syndrome, RNA binding protein, gene name

## Introduction

In the early days of gene discoveries, the denominations in initial reports were often imprecise, restrictive or uninformative. Indeed, genes were usually originally named in reference to the context of their discovery, according to the specific tissue, cell type, cellular function, disease or phenotypic outcome under scrutiny. As a result, it was not uncommon that the same gene would have several names, simply because different researchers were working on the same gene, albeit in distinct contexts. Consequently, despite gene names harmonization, which coincided with the adoption of official gene symbols used in databases, most genes still exhibit a handful of aliases. Also, the official alias was generally chosen on historical grounds rather than as a reflection of the actual functions of the gene. More recently, several genes were renamed as their historical name was either unprecise and/or vector of negative connotations. Renaming the *FMR1* gene has recently been the object of such debate in the community (Herring et al., 2020).

## The specific case of renaming the FMR1 gene involved in fragile X syndrome

Since its cloning in 1991 (Verkerk et al., 1991), the official name of the gene mutated in Fragile X Syndrome (FXS) has been *Fragile X Mental Retardation 1* (official gene symbol *FMR1*), as it was the first of a long series of X-linked genes associated with intellectual deficiency, termed at that time mental retardation. FXS is a leading cause of developmental delay, inherited intellectual disability, and the most frequent monogenic cause of autism spectrum disorder (ASD) (Kaufmann et al., 2017). FXS is caused by abnormal expansions of CGG triplets (*n* > 200 repeats) in the untranslated region (5′-UTR) of the X-linked *FMR1* gene, which turn off its expression (Verkerk et al., 1991). Over the past two decades, while the general population exhibit in average 30 CGG repeats, premutations between 50 and 200 repeats in the *FMR1* gene have been associated with Fragile X Premutation Associated Conditions (FXPAC), a set of conditions with no intellectual disability (Hagerman and Hagerman, 2016; Johnson et al., 2020). Also, it is important to note that mutations in the *FMR1* gene are incompletely penetrant, and some individuals, in particular females bearing abnormal CGG repeat expansions will exhibit no signs of FXS or FXPAC (Johnson et al., 2020). As our society becomes more inclusive and recommends not to stigmatize individuals with intellectual deficiency, the term mental retardation has been progressively banned from scientific or clinical reports. Also, since *FMR1* mutations are not necessarily accompanied by intellectual deficiency, calling it based on this single phenotypic trait was judged non-inclusive (Herring et al., 2020). Upon a suggestion of the European Fragile X Network that received support from Fragile X Societies, it was petitioned to change the definition of the *FMR1* acronym as well as of its encoded protein FMRP, by modifying the meaning of the last letters (Herring et al., 2020). As a result, recently, the HUGO Gene Nomenclature Committee agreed to rename the *FMR1* gene with the more inclusive name of *Fragile X Messenger Ribonucleoprotein 1* (HGNC, 2021; Bruford and On behalf of the Hugo Gene Nomenclature Committee, 2022).

## Discussion

We agree that removing “mental retardation” was necessary and keeping the original acronym *FMR1* was essential for database nomenclature issues. However, we wish to point that the new name “*Fragile X Messenger Ribonucleoprotein”* does not reflect the pleiotropic functions of *FMR1* gene that three decades of research have contributed to unravel. The letter M now standing for “messenger” and the R for “ribonucleoprotein. First, the choice of “messenger” for M does not reflect the fact that FMRP, the *FMR1* encoded protein, not only binds messenger RNA in the translation machinery to modulate translation (Corbin et al., 1997; Khandjian et al., 2004; Stefani et al., 2004; Darnell et al., 2011), but also micro-RNA, piwi-RNA, and lncRNA (Caudy et al., 2002; Guo et al., 2018; Lannom et al., 2021). Therefore, its principal functional category is RNA-binding protein, although FMRP not only binds RNA, but also chromatin where it intervenes in DNA repair processes (Alpatov et al., 2014; Chakraborty et al., 2020). Second, the abbreviation, MRP for “messenger-ribonucleoprotein” is insufficiently precise, a more adequate term would have been “messenger-binding”. Third, FMRP not only binds nucleic acids, but also a diversity of partner proteins within large protein complexes [Figure 10A in Dury et al. (2013), Brown et al. (2010)]. These multiple protein-protein interactions likely modulate FMRP affinity to RNA (Bechara et al., 2007). In addition, it should be recalled that FMRP, besides being a critical component of neuronal granules, which travel in the neuronal arborization to deliver RNA at the synapse (El Fatimy et al., 2016), is also a molecular adaptor or a bridge between these granules and microtubule elements (Davidovic et al., 2007; Dictenberg et al., 2008). For all these reasons, assigning the term “messenger ribonucleoprotein” in *FMR1* full name *de facto* restricts its biological role and do not reflect the full spectrum of the 26 functional categories assigned to *FMR1* in the Gene Ontology (GO) repository. As a matter of fact, GO terms associated to *FMR1* notably encompass: Nucleic acid binding (GO:0003676), RNA-binding (GO:0003729), miRNA-binding (GO:0035198), siRNA binding (GO:0035197), ribosome binding (GO:0043022), methylated histone binding (GO:0035064), to microtubule binding (GO:0008017), protein binding (GO:0001948), and protein homodimerization activity (GO:0042803). These multifaceted functions should be reflected in *FMR1* new alias.

**FIGURE 1:**
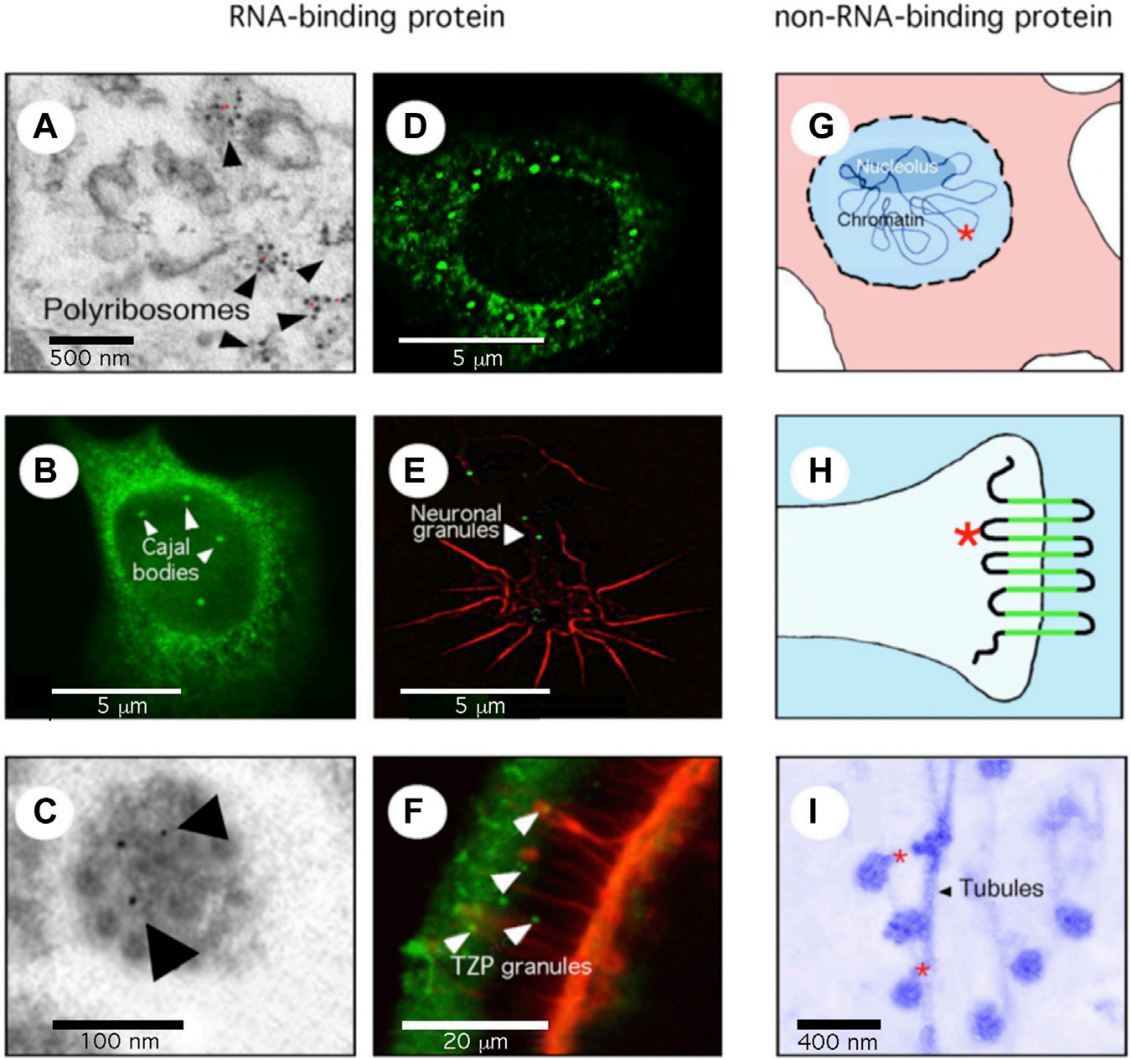
FMRP is a multifunctional protein. **(A)** the great majority of FMRP is associated with the translation machinery. Electron microscopy (EM) micrography showing FMRP (red dots) on polyribosomes. **(B)** A minor fraction of FMRP, in this case the nuclear isoform 6, is detected in Cajal Bodies, site of splicing of pre-messenger RNA. **(C)** FMRP is present in compacted granules containing mRNA, travelling in neuronal dendrites/axons as seen by EM and immuno-gold labelling (indicated by arrows). **(D)** FMRP relocalizes to stress granules after arsenite treatments. **(E)** FMRP (green) in compacted granules delivered locally at the axonal growth cone (red). **(F)** FMRP (green) is present in RNA-granules translocated from cumulus cells to the oocytes through transzonal projections (red). **(G)** FMRP (red star) is associated with chromatin and is involved in DNA repair. **(H)** FMRP (red star) associates with ion channels at the synapse. **(I)** FMRP (red dots) is a molecular adaptor between repressed granules and microtubules through kinesin interaction in the neuronal arborization.

As for the protein name, the UniProt database has chosen a name totally unrelated to the acronym as it is now termed “Synaptic functional regulator *FMR1*” (https://www.uniprot.org/uniprot/Q06787). This is also clearly misleading and restrictive in terms of functions for FMRP in other cell types than neurons and outside the brain. The synaptic compartment containing only a minor fraction of FMRP, and its functions are in no way restricted to the synapse. As a matter of fact, FMRP is expressed in all tissues of the body, albeit at different levels (Khandjian et al., 1998, see also Figure 1) to the exception of striated muscle (Khandjian et al., 1998). As a consequence, in light of the 30 years of research in the Fragile X field, neither the HUGO name for the *FMR1* gene, nor the UniProt name for FMRP appear adequate since they do not actually reflect their pleiotropic functions.

Consequently, we wish to propose here a more inclusive full name for *FMR1*. Keeping intact the *FMR1* acronym, we propose the F to remain as in Fragile X, but that M stands for multifunctional, R for RNA-binding, the main functional category assigned to FMRP, and obviously in the case of its encoded product, P for protein. We believe that “Fragile X Multifunctional RNA-binding Protein” is a more naturalistic full name which captures best the multifaceted functions of FMRP unravelled in the course of 30 years of research in the field.
